# Incidence, predictors and prognostic implications of positive circumferential resection margin in colon cancer: A retrospective study in a Chinese high-volume cancer center

**DOI:** 10.3389/fonc.2022.871570

**Published:** 2022-09-20

**Authors:** Dakui Luo, Jing Li, Weijing He, Yufei Yang, Sanjun Cai, Qingguo Li, Xinxiang Li

**Affiliations:** ^1^ Department of Colorectal Surgery, Fudan University Shanghai Cancer Center, Shanghai, China; ^2^ Department of Oncology, Shanghai Medical College, Fudan University, Shanghai, China; ^3^ Department of CyberKnife Center, Huashan Hospital, Fudan University, Shanghai, China

**Keywords:** circumferential resection margin, colon cancer, surgery, logistic analysis, FUSCC

## Abstract

Positive circumferential resection margin (CRM) was associated with a higher recurrence rate and worse survival in rectal cancer. Predictors of CRM in rectal cancer have widely been investigated. Our study aims to determine the incidence, predictors and prognostic implications of positive CRM following colon cancer (CC) surgery in a Chinese high-volume cancer center. The clinicopathological features and oncological outcomes of CC patients undergoing surgery between January 2008 and December 2018 were identified from Fudan University Shanghai Cancer Center database. Positive CRM was defined as resection margin ≤1 mm. A total of 5268 stage I-IV CC patients were identified in our study, 108 (2.05%) of whom had positive CRM. Multivariate logistic analysis found that advanced N stage, distant metastases and poorly differentiated tumor had increased risk of positive CRM. After propensity score matching, the 5-year overall survival rates of the patients with positive and negative CRM were 33.2% and 39.8% (P=0.005), respectively. Multivariable COX regression model showed that positive CRM was an independent prognostic factor for OS in CC patients. The overall rate of positive CRM in our center is lower than that in western population. Several adverse pathological parameters deserve more attention to identify CC patients at a high risk of positive CRM. Adoption of appropriate surgical techniques and multidisciplinary treatment planning are expected to improve oncological outcomes for high selected CC patients with “high-risk” CRM involvement.

## Introduction

Surgical resection with total mesorectal excision (TME) has been established as a standard care for curative rectal cancer (1). Complete TME requires a clear circumferential resection margin (CRM), defined as resection margin ≥1 mm from the CRM on pathologic specimen (2). Positive CRM represents incompleteness of resection, which is a major determinant of local relapse (3). Evidence indicated that positive CRM after rectal cancer surgery predicted poor prognosis (4–6). As a result, the status of CRM has been widely employed as an important indicator for surgical quality in rectal cancer.

Several sporadic studies reported the incidence, risk factors and prognostic implications of positive CRM in colon cancer with great heterogeneity (7–11). These studies provided important insights into CRM of colon cancer in western countries. We wonder if there are distinct characteristics of positive CRM after colon cancer surgery between China and western countries.

Therefore, our study aimed to determine the rate of positive CRM and identify risk factors of positive CRM following colon cancer surgery at a university teaching center in China.

## Method

### Study population

The data of 7231 CC patients who received resection of primary lesion in Fudan University Shanghai Cancer Center (FUSCC) (Shanghai, China) during the period January 2008 through December 2018 were reviewed. Dataset of colorectal cancer patients in FUSCC was built prospectively.The information of colorectal cancer patients treated in FUSCC was recorded since January 2008. All patients who received resection of colorectal cancer were included in the database. In general, the following variables were collected: age at diagnosis; gender; year at diagnosis; tumor location; date of surgery; surgical procedures; neoadjuvant therapy; detailed data regarding pathology; survival data. Medical records review, telephone visits and death registry data linkage were carried out for collecting endpoint data. These data were collected by staffs of Clinical Statistics Center, FUSCC.

Of these, 5268 cases had definite status of CRM. The following variables were extracted from FUSCC database: status of CRM; age at diagnosis; gender; year at diagnosis; tumor location; surgical procedures; neoadjuvant chemotherapy; histologic type; differentiation; pathological stage; perineural invasion; vascular invasion; survival data. The study protocol was approved by the ethics committee of FUSCC.

### Pathological evaluation

Positive CRM involvement was defined as tumor presence in distance ≤1 mm from the nonperitonealized surface of resection, whereas negative CRM was defined as >1 mm in distance. Patients with no/unknown CRM information were excluded from our study. Pathological examination of the specimen was evaluated by two independent gastrointestinal subspecialty pathologists. When there was disagreement, a third pathologist would reexamine the sample and the majority of the opinion was the final opinion.

### Postoperative follow-up

Medical record reviews, telephonic visit follow-ups and death registry data linkage were employed for collecting survival data. The last follow-up date was September. 30, 2020.

### Statistical analysis

The statistical analyses were performed using SPSS version 25.0 (SPSS, Chicago, Illinois, USA) and R (https://www.r-project.org/, version 4.1.1). The chi-squared test was used for thecomparison of categorical variables. Univariate and multivariate binary logistic regression *via* the entering method was performed to explore variables predictive of positive CRM. Survival curves were estimated according to the Kaplan–Meier method and compared by the log-rank test. To minimize the inherent selection bias, propensity score matching (PSM) at a 1:2 ratio was performed to compare OS. The PSM model was based upon age at diagnosis, gender, year at diagnosis, tumor location, histologic type, differentiation, pathological stage, perineural invasion, vascular invasion. Multivariate analysis using Cox regression was performed to identify the independent prognostic factors. A p-value of <0.05 was considered statistically significant.

## Results

A total of 5268 stage I-IV CC patients were identified in our study. Demographic and pathological characteristics are shown in **Table 1**. Overall, there were 108 (2.05%) CRM positive patients and 5160 CRM negative patients with a slight decline in CRM positivity observed from 2008-2013 (2.2%) to 2014-2018 (1.9%). Positive CRM was identified in 1.9% of T3 patients and 2.8% of T4 patients, respectively.

**Table 1:** Comparison of clinicopathological features according to CRM status before PSM.

	Negative CRM (n=5160)	Positive CRM (n=108)	P value
**Age (years)**			0.512
<60	2416 (46.8)	54 (50.0)	
≥60	2744 (53.2)	54 (50.0)	
**Sex**			0.477
Male	2977 (57.7)	66 (61.1)	
Female	2183 (42.3)	42 (38.9)	
**Years**			0.564
2008-2013	2722 (52.8)	60 (55.6)	
2014-2018	2438 (47.2)	48 (44.4)	
**Location**			0.024
Left side	2378 (46.1)	38 (35.2)	
Right side	2782 (53.9)	70 (64.8)	
**Surgical procedures**			0.603
Laparoscopic	711 (13.8)	13 (12.0)	
Open	4449 (86.2)	95 (88.0)	
**Neoadjuvant therapy**			0.085
No	210 (4.1)	8 (7.4)	
Yes	4950 (95.9)	100 (92.6)	
**Histologic type**			<0.001
Adenocarcinoma	4293 (83.2)	73 (67.6)	
Mucinous	748 (14.5)	26 (24.1)	
Signet ring cell	94 (1.8)	9 (8.3)	
Unknown	25 (0.5)	0 (0.0)	
**Differentiation**			<0.001
Poor	1342 (26.0)	56 (51.9)	
Moderate	3468 (67.2)	44 (40.7)	
Well	107 (2.1)	1 (0.9)	
Unknown	243 (4.7)	7 (6.5)	
**T stage**			<0.001
Tx, 0-2	894 (17.3)	1 (0.9)	
T3	1525 (29.6)	29 (26.9)	
T4	2741 (53.1)	78 (72.2)	
**N stage**			<0.001
N0	2695 (52.2)	14 (13.0)	
N1	1581 (30.6)	38 (35.2)	
N2	884 (17.1)	56 (51.9)	
**M stage**			<0.001
0	4319 (83.7)	57 (52.8)	
1	841 (16.3)	51 (47.2)	
**AJCC stage**			<0.001
0-I	598 (11.6)	0 (0.0)	
II	1807 (35.0)	12 (11.1)	
III	1813 (35.1)	45 (41.7)	
IV	841 (16.3)	51 (47.2)	
Unknown	101 (2.0)	0 (0.0)	
**Perineural invasion**			<0.001
Negative	3980 (77.1)	57 (52.8)	
Positive	1180 (22.9)	51 (47.2)	
**Vascular invasion**			<0.001
Negative	3781 (73.3)	46 (42.6)	
Positive	1379 (26.7)	62 (57.4)	

CRM, circumferential resection margin; PSM, propensity score matching.

### Risk factors associated with positive CRM

Univariate logistic analysis showed that patients with positive CRM were more likely to have right-sided cancer [right-sided versus left-sided, odds ratio (OR): 1.575,95% confidence interval (CI): 1.057-2.346, P=0.026], to have disease of mucinous or signet-ring cell histology (mucinous versus adenocarcinoma, OR: 2.044, 95% CI: 1.298-3.220, P=0.002; signet-ring cell versus adenocarcinoma, OR: 5.631, 95% CI: 2.735-11.590, P<0.001), to have more poorly differentiated tumors (moderate versus poor, OR: 0.304, 95% CI: 0.204-0.454, P<0.001), to be diagnosed with a more advanced stage (N1 versus N0, OR: 4.627, 95% CI: 2.499-8.566, P<0.001; N2 versus N0, OR: 12.195, 95% CI: 6.756-22.011, P<0.001; M1 versus M0, OR: 4.595, 95% CI: 3.127-6.752, P<0.001; IV versus II+III, OR: 3.851, 95% CI: 2.620-5.661, P<0.001) and to have perineural (positive versus negative, OR: 3.018, 95% CI: 2.057-4.428, P<0.001) or vascular invasion (positive versus negative, OR: 3.696, 95% CI: 2.511-5.438, P<0.001).

Multivariate logistic analysis found that advanced N stage (N1 versus N0, OR: 2.563, 95% CI: 1.330-4.939, P=0.005; N2 versus N0, OR: 4.298, 95% CI: 2.147-8.601, P<0.001), distant metastases (M1 versus M0, OR: 2.347, 95% CI: 1.527-3.608, P<0.001) and poorly differentiated tumor had increased risk of positive CRM (poor versus moderate, OR:1.710, 95% CI: 1.071-2.729) **Table 2**.

**Table 2:** Univariable and multivariable logistic regression model for factors predictive of positive CRM after colon cancer resection.

	Univariable		Multivariable	
	OR (95%CI)	P	OR (95%CI)	P
**Age (years)**	0.983 (0.968-0.998)	0.025	0.994 (0.979-1.010)	0.468
**Sex**				
Male	Reference		Reference	
Female	0.868 (0.587-1.283)	0.477	0.804 (0.531-1.219)	0.304
**Years**				
2008-2013	Reference		Reference	
2014-2018	0.893 (0.609-1.311)	0.564	1.262 (0.728-2.190)	0.407
**Location**				
Left side	Reference		Reference	
Right side	1.575 (1.057-2.346)	0.026	1.330 (0.869-2.035)	0.189
**Surgical procedures**				
Open	Reference			
Laparoscopic	0.856 (0.477-1.537)	0.603	1.026 (0.548-1.923)	0.936
**Neoadjuvant therapy**				
No	Reference		Reference	
Yes	1.886 (0.906-3.926)	0.090	1.600 (0.719-3.558)	0.249
**Histologic type**				
Adenocarcinoma	Reference		Reference	
Mucinous	2.044 (1.298-3.220)	0.002	1.342 (0.779-2.310)	0.289
Signet ring cell	5.631 (2.735-11.590)	<0.001	1.845 (0.771-4.414)	0.169
**Differentiation**				
Moderate	Reference		Reference	
Poor	3.289 (2.205-4.906)	<0.001	1.710 (1.071-2.729)	0.025
Well	0.737 (0.101-5.396)	0.764	3.308 (0.419-26.136)	0.257
**T stage**				
T3	Reference		Reference	
T4	1.496 (0.973-2.302)	0.067	1.677 (0.912-3.080)	0.096
**N stage**				
N0	Reference		Reference	
N1	4.627 (2.499-8.566)	<0.001	2.563 (1.330-4.939)	0.005
N2	12.195 (6.756-22.011)	<0.001	4.298 (2.147-8.601)	<0.001
**M stage**				
0	Reference		Reference	
1	4.595 (3.127-6.752)	<0.001	2.347 (1.527-3.608)	<0.001
**Perineural invasion**				
Negative	Reference		Reference	
Positive	3.018 (2.057-4.428)	<0.001	1.245 (0.807-1.921)	0.322
**Vascular invasion**				
Negative	Reference		Reference	
Positive	3.696 (2.511-5.438)	<0.001	1.319 (0.827-2.104)	0.246

### Survival analyses

**Figure 1:**
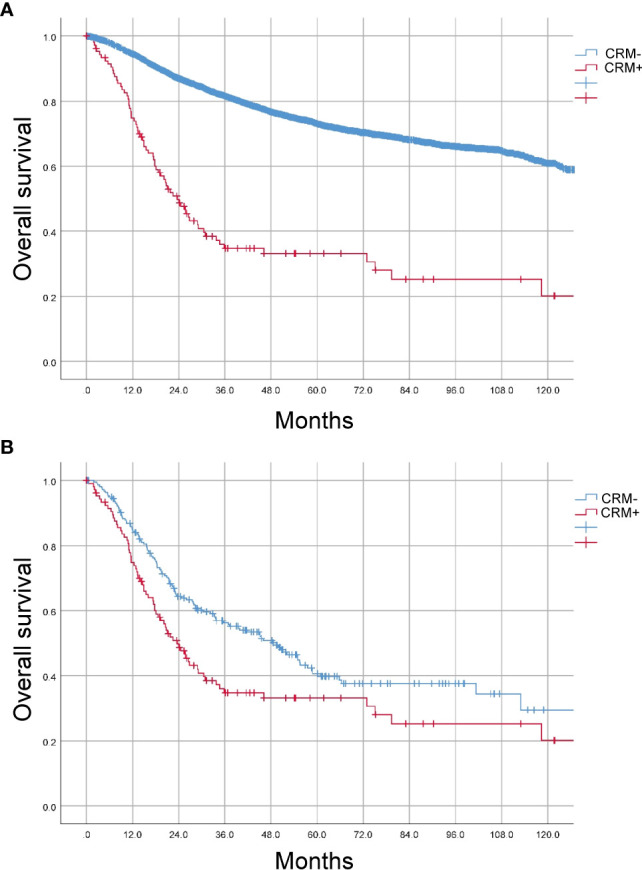
Prognostic value of CRM status in CC patients. **(A)** Kaplan-Meier (K-M) curves were plotted before PSM. **(B)** Kaplan-Meier (K-M) curves were plotted after PSM.

Next, we explored the prognostic value of CRM status in colon cancer. The 5-year overall survival (OS) rates of the patients with positive and negative CRM were 33.2% and 73.0% (P<0.001, **Figure 1A**). After PSM, the baseline characteristics between two groups were comparable (**Supplementary Table 1**). The 5-year OS rates of the patients with positive and negative CRM were 33.2% and 39.8% (P=0.005), respectively (**Figure 1B**). In univariate Cox analysis, age, tumor location, neoadjuvant therapy, histologic type, grade, pathological stage, perineural or vascular invasion and CRM were associated with OS in entire cohort. After adjusting for known confounders, patients with positive CRM had a 105% increase in the hazard of death (HR: 1.2.050, 95% CI: 1.584-2.653, P<0.001). Additionally, age, tumor location, adoption of neoadjuvant therapy, histologic type, grade, pathological stage, perineural or vascular invasion were independent risk factors for OS in colon cancer patients who received primary surgery (**Table 3**).

**Table 3:** Univariate and multivariate Cox analyses of the factors for overall survival in colon cancer.

Variable	Univariate analysis		Multivariate analysis	
	HR (95% CI)	P value	HR (95% CI)	P value
**Age (years)**	1.016 (1.011-1.020)	<0.001	1.031 (1.026-1.036)	<0.001
**Sex**				
Male	Reference		NI	
Female	1.016 (0.915-1.129)	0.766		
**Years**				
2008-2013	Reference		NI	
2014-2018	0.984 (0.881-1.099)	0.773		
**Location**				
Left side	Reference		Reference	
Right side	1.181 (1.063-1.312)	0.002	1.145 (1.027-1.278)	0.015
**Surgical procedures**				
Open	Reference		Reference	
Laparoscopic	0.851 (0.716-1.011)	0.067	0.932 (0.777-1.119)	0.452
**Neoadjuvant therapy**				
No	Reference		Reference	
Yes	2.519 (2.058-3.083)	<0.001	1.642 (1.310-2.058)	<0.001
**Histologic type**				
Adenocarcinoma	Reference		Reference	
Mucinous	1.253 (1.090-1.440)	0.001	1.174 (1.001-1.376)	0.049
Signet ring cell	2.762 (2.106-3.622)	<0.001	1.863 (1.378-2.521)	<0.001
Unknown	/		/	
**Differentiation**				
Moderate	Reference		Reference	
Poor	1.839 (1.647-2.053)	<0.001	1.256 (1.106-1.425)	<0.001
Well	0.666 (0.423-1.050)	0.080	1.306 (0.820-2.082)	0.261
Unknown	/		/	
**T stage**				
Tx, 0-2	Reference		Reference	
T3	2.550 (2.053-3.168)	<0.001	1.228 (0.969-1.555)	0.089
T4	2.995 (2.455-3.654)	<0.001	1.421 (1.143-1.765)	0.002
**N stage**				
N0	Reference		Reference	
N1	2.693 (2.361-3.071)	<0.001	1.885 (1.633-2.177)	<0.001
N2	5.735 (5.020-6.551)	<0.001	3.077 (2.616-3.618)	<0.001
**M stage**				
0	Reference		Reference	
1	6.471 (5.811-7.205)	<0.001	4.469 (3.971-5.030)	<0.001
**Perineural invasion**				
Negative	Reference		Reference	
Positive	2.256 (2.023-2.515)	<0.001	1.332 (1.183-1.500)	<0.001
**Vascular invasion**				
Negative	Reference		Reference	
Positive	2.736 (2.464-3.038)	<0.001	1.279 (1.131-1.446)	<0.001
**CRM**				
Negative	Reference		Reference	
Positive	4.199 (3.295-5.352)	<0.001	2.050 (1.584-2.653)	<0.001

CRM, circumferential resection margin.

## Discussion

The incidence, risk factors and prognostic implications of positive CRM in CC have been well explored in western populations. However, there is a lack of data in the Chinese population. Our current study represents the largest series on status of CRM in CC patients who received resection of primary lesion in a Chinese high-volume cancer center. Our study found that positive CRM was identified in 2.05% of general Chinese CC patients and was associated with several adverse pathologic characteristics. Furthermore, we found that positive CRM predicted worse prognosis in these patients.

The published data presented a wide variation in the incidence of positive CRM in CC surgery. Amri et al. retrospectively analyzed 984 patients with surgically treated CC and identified 52 (5.3%) patients with a positive CRM (8). Another study included 189,343 locally advanced CC patients from the National Cancer Database and demonstrated that positive CRM was identified in 9% of stage II and 12% of stage III CC patients (9). A SEER-based study reported that 20.5% patients were CRM-positive following resection of CC in general population, which represented the highest rate of positive CRM in CC (11) among the listed studies. Recently, a cohort study of 170,022 colon cancer cases in U.S. hospitals revealed that positive CRM occurred in 11.6% of this population. The overall rate of positive CRM in United States is high with unexpected variation across hospitals (10). These studies were conducted in western countries. The rate of positive CRM was observed in 2.05% of general CC patients in our study. This is lower than that of positive CRM reported in previous studies. It is possible that these findings are influenced by review of pathologic specimens. Besides that, evidence indicated that the hospital volume was a predictor of positive CRM in CC surgery (10). Treatment by high-volume surgeons at high volume centers could decrease the risk of CRM involvement. The obvious difference could be caused by different populations with distinctive biology. Evidence indicated that the plane of surgery achieved has an essential effect on status of CRM and local recurrence in rectal cancer (12). Although aggressive tumor biology is associated with the likelihood of positive CRM, the low rate of positive CRM represents the optimal management quality of colon cancer in our center. The excellent outcomes are a result of individual surgeon experience or inherent resources available at high-volume centers.

As expected, high-risk pathological features, such as tumor stage, grade and perineural invasion were independently associated with CRM involvement in our study. These results can contribute to distinguish between poor technique and aggressive tumor biology in patients with poor surgical outcomes. The strongest predictor of risk for positive CRM was the lymph nodes involvement. Besides that, the adoption of MR to predict CRM involvement has been accurate in tailoring neoadjuvant chemoradiotherapy in rectal cancer (13). However, the use of neoadjuvant therapy to intensify treatment is less well established in CC. Identifying these risk factors for CRM involvement of CC could contribute to optimize surgical quality and improve outcomes. It is undeniable that aggressive tumor biology still increases the risk of positive CRM despite the optimal surgical procedure. Intensive preoperative therapy for these patients merits further investigation.

Evidence indicated that positive CRM predicted poor prognosis. Khan et al. found that the overall relapse rate was 18.9% in R0 and 55.5% in R1 resections of CC (14). Similarly, Goffredo et al. reported that positive CRM was associated with significantly lower overall survival on both univariate and multivariable Cox analysis. In agreement with previous results, the 5-year overall survival rates of the CC patients with positive and negative CRM were 33.2% and 39.8% (P=0.005) in our study. Over the past 30 years, rectal cancer surgery has been standardized by TME, which offers the lowest risk of local recurrence and the excellent survival benefits in patients with rectal cancer. In CC surgery, complete mesocolic excision (CME) with central vascular ligation is preferred especially in western counties while D3 dissection has been widely adopted in Japan (15). It is necessary for surgeons to improve surgical techniques in CC resection, decreasing the risk of CRM involvement andlocal recurrence, and optimizing oncological outcomes.

Neoadjuvant therapy may achieve tumor downstaging, which in turn may facilitate the chance of radical surgery and was expected to decrease the overall risk of CRM involvement. However, our study did not observe the protective role of neoadjuvant therapy. This may be explained by aggressive tumor biology, lack of downstaging and tumor resistance to chemotherapy.

CRM is a widely accepted indicator for quality control program. Surgeons and pathologists of multidisciplinary treatment planning and training are expected to improve surgical quality. Besides that, providing feedback to surgeons about their rates of CRM positivity contributed to a decline in rates of CRM. For patients with “high-risk” CRM involvement, the surgery should be performed by senior surgeons.

We recognize several limitations in this work. Heterogeneity in surgical technique, specimen processing and pathologic interpretation are inevitable. Especially, a large majority of the patients received open surgery while only 13.8% patients received laparoscopic surgery. However, no difference was observed between open and laparoscopic surgery in terms of CRM positivity. In fact, the patients were recruited from January 2008 to December 2018 in our present study. In recent years, laparoscopic surgery has been adopted as a current daily practice in management of CC patients. Additionally, some clinically relevant variables and detailed information regarding relapse were not collected in our database. Finally, we anticipated hospital-level variation in CRM positivity. In consideration that our analysis was restricted to a Chinese high-volume cancer center, the results should thus be extended to general Chinese population with caution.

In conclusion, overall rate of positive CRM in our single center is low for CC patients. Several adverse pathologic characteristics have been identified as independent risk factors. Positive CRM has an adverse impact on overall survival. Although aggressive tumor biology is a critical factor, adoption of appropriate surgical techniques and multidisciplinary treatment planning are expected to improve oncological outcomes for CC patients with “high-risk” CRM involvement. More studies are needed to establish the role of neoadjuvant therapy in this subgroup.

## Data availability statement

The raw data supporting the conclusions of this article will be made available by the authors, without undue reservation.

## Ethics statement

The studies involving human participants were reviewed and approved by the Ethical Committee and Institutional Review Board of the FUSCC. The patients/participants provided their written informed consent to participate in this study.

## Author contributions

XL, QL and DL conceived this study. DL, JL and SC improved the study design and contributed to the interpretation of results. WH and YY collected the data. DL performed data processing and statistical analysis. DL and XL wrote the manuscript. QL revised the manuscript. All authors approved the final version.

## Funding

This work was supported by the National Natural Science Foundation of China (Grant NO. 81972260; NO. 82103259), Shanghai Municipal Natural Science Foundation(21ZR1414400), and Shanghai Medical Innovation Research Project(22Y11907600). The funders had no role in the study design, data collection and analysis, decision to publish, or preparation of the manuscript.

## Acknowledgments

The authors acknowledge the efforts of the staff from Fudan University Shanghai Cancer Center in the management of the FUSCC database.

## Conflict of interest

The authors declare that the research was conducted in the absence of any commercial or financial relationships that could be construed as a potential conflict of interest.

## Publisher’s note

All claims expressed in this article are solely those of the authors and do not necessarily represent those of their affiliated organizations, or those of the publisher, the editors and the reviewers. Any product that may be evaluated in this article, or claim that may be made by its manufacturer, is not guaranteed or endorsed by the publisher.

## Supplementary material

Supplementary Table 1 Comparison of clinicopathological features according to CRM status after PSM.
