# Supplementary material for: Incidence, predictors and prognostic implications of positive circumferential resection margin in colon cancer: A retrospective study in a Chinese high-volume cancer center
Source: Front Oncol. 2022 Sep 20;12:871570. doi: 10.3389/fonc.2022.871570 (PMC9530821; doi:10.3389/fonc.2022.871570)
Supplement: Supplementary Table 1 — Comparison of clinicopathological features according to CRM status after PSM. [file Table_1.docx]

**Supplementary Table 1** Comparison of clinicopathological features according to CRM status after PSM.

|  | Negative CRM (n=216) | Positive CRM (n=108) | P value |
| --- | --- | --- | --- |
| **Age (years)** |  |  | 0.529 |
| <60 | 100 (46.3) | 54 (50.0) |  |
| ≥60 | 116 (53.7) | 54 (50.0) |  |
| **Sex** |  |  | 1.000 |
| Male | 132 (61.1) | 66 (61.1) |  |
| Female | 84 (38.9) | 42 (38.9) |  |
| **Years** |  |  | 0.479 |
| 2008-2013 | 111 (51.4) | 60 (55.6) |  |
| 2014-2018 | 105 (48.6) | 48 (44.4) |  |
| **Location** |  |  | 0.466 |
| Left side | 85 (39.4) | 38 (35.2) |  |
| Right side | 131 (60.6) | 70 (64.8) |  |
| **Surgical procedures** |  |  | 1.000 |
| Laparoscopic | 26 (12.0) | 13 (12.0) |  |
| Open | 190 (88.0) | 95 (88.0) |  |
| **Neoadjuvant therapy** |  |  | 0.416 |
| No | 194 (89.8) | 100 (92.6) |  |
| Yes | 22 (10.2) | 8 (7.4) |  |
| **Histologic type** |  |  | 0.160 |
| Adenocarcinoma | 164 (75.9) | 73 (67.6) |  |
| Mucinous | 39 (18.1) | 26 (24.1) |  |
| Signet ring cell | 10 (4.6) | 9 (8.3) |  |
| Unknown | 3 (1.4) | 0 (0.0) |  |
| **Differentiation** |  |  | 0.129 |
| Poor | 93 (43.1) | 56 (51.9) |  |
| Moderate | 113 (52.3) | 44 (40.7) |  |
| Well | 0 (0.0) | 1 (0.9) |  |
| Unknown | 10 (4.6) | 7 (6.5) |  |
| **T stage** |  |  | 0.314 |
| T2 | 0 (0.0) | 1 (0.9) |  |
| T3 | 65 (30.1) | 29 (26.9) |  |
| T4 | 151 (69.9) | 78 (72.2) |  |
| **N stage** |  |  | 0.969 |
| N0 | 29 (13.4) | 14 (13.0) |  |
| N1 | 73 (33.8) | 38 (35.2) |  |
| N2 | 114 (52.8) | 56 (51.9) |  |
| **M stage** |  |  | 0.937 |
| 0 | 113 (52.3) | 57 (52.8) |  |
| 1 | 103 (47.7) | 51 (47.2) |  |
| **AJCC stage** |  |  | 0.984 |
| II | 25 (11.6) | 12 (11.7) |  |
| III | 88 (40.7) | 45 (41.7) |  |
| IV | 103 (47.7) | 51 (47.2) |  |
| **Perineural invasion** |  |  | 0.753 |
| Negative | 110 (50.9) | 57 (52.8) |  |
| Positive | 106 (49.1) | 51 (47.2) |  |
| **Vascular invasion** |  |  | 0.937 |
| Negative | 91 (42.1) | 46 (42.6) |  |
| Positive | 125 (57.9) | 62 (57.4) |  |

CRM, circumferential resection margin; PSM, propensity score matching.
